# Extracellular vesicle-based delivery systems for nucleic acid therapeutics

**DOI:** 10.1016/j.omtn.2026.102870

**Published:** 2026-02-17

**Authors:** Xiaoqiong Zhang, Xuhan Liu, Qing Zhou, Kai Yao

**Affiliations:** 1 Institute of Visual Neuroscience and Stem Cell Engineering, College of Life Sciences and Health, Wuhan University of Science and Technology, Wuhan 430065, China; 2 Guangdong Provincial Key Laboratory of Chinese Medicine Ingredients and Gut Microbiomics, Institute for Inheritance-Based Innovation of Chinese Medicine, Marshall Laboratory of Biomedical Engineering, School of Pharmacy, Shenzhen University Medical School, Shenzhen University, Shenzhen 518055, China; 3 The Center for Biomedical Research, NHC Key Laboratory of Respiratory Diseases, Department of Respiratory and Critical Care Medicine, Tongji Hospital, Tongji Medical College, Huazhong University of Sciences and Technology, Wuhan 430030, China; 4 Xianning Medical College, Hubei University of Science & Technology, Xianning 437199, China

**Keywords:** MT: Delivery Strategies, nucleic acid therapeutics, extracellular vesicles, drug delivery systems, nucleic acid loading, administration routes

## Abstract

Nucleic acid-based therapeutics, which involve the manipulation of genetic materials to treat or prevent diseases, have gained considerable attention, leading to the approval of medicines such as COVID-19 vaccines, patisiran (Onpattro), and nusinersen (Spinraza). However, their clinical application is hindered by challenges such as nuclease degradation, poor biodistribution, limited cellular uptake, and inefficient endosomal escape. Extracellular vesicles (EVs), which are natural nanoscale drug delivery systems derived from various eukaryotic and prokaryotic cells, offer a safe, efficient, specifically targeted, and non-pathogenic method for nucleic acid delivery. In this review, we summarize the classical methods and the latest research advances in EV preparation and nucleic acid loading. Additionally, we review the primary administration routes for nucleic acid-loaded EVs, such as intravenous, local, oral, intranasal, and inhalation delivery. By addressing these aspects, this review aims to guide the optimal design and clinical application of nucleic acid-loaded EVs.

## Graphical abstract

**Figure:**
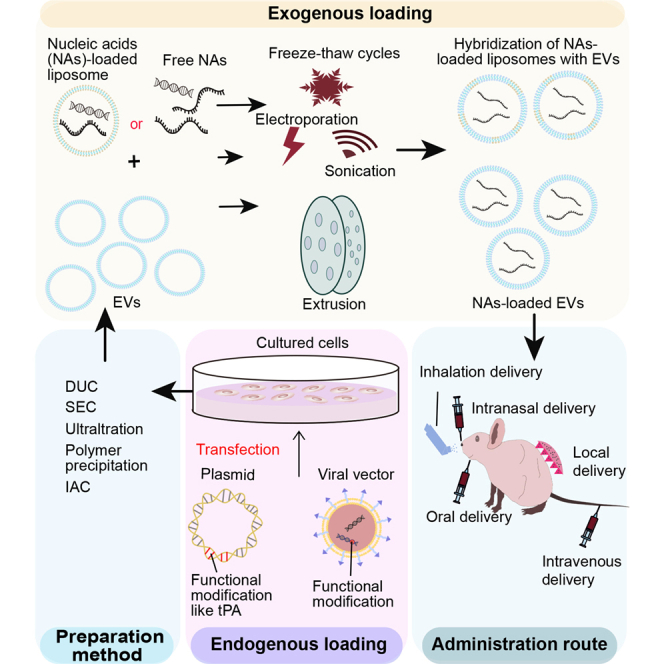

## Teaser

Zhou and colleagues review methods in EV preparation and nucleic acid loading, discussing administration routes for nucleic acid-loaded EVs, including intravenous, local, oral, intranasal, and inhalation delivery to guide optimal design and clinical application of nucleic acid-loaded EVs.

## Introduction

Gene therapy represents a groundbreaking strategy for the treatment and prevention of human diseases by modifying genetic materials to regulate gene expression. By delivering nucleic acids such as small interfering RNAs (siRNAs), microRNAs (miRNAs), antisense oligonucleotides (ASOs), messenger RNAs (mRNAs), clustered regularly interspaced short palindromic repeat (CRISPR)-Cas systems, or functional DNA copies, gene therapy can be employed to either promote the expression of beneficial genes or suppress the activity of pathogenic genes.^1^^,^^2^^,^^3^ In general, mRNA- and DNA-based approaches are applied to increase or restore gene expression, whereas siRNAs, miRNAs, ASOs and CRISPR-Cas systems are typically utilized to downregulate or disrupt disease-associated genes. The mRNAs have emerged as a promising tool for therapeutic applications, including protein replacement therapy, vaccines, and gene editing. Once delivered into the cytoplasm, mRNAs are translated into proteins by ribosomes, thus avoiding the need to cross the nuclear barrier.^1^ The siRNAs are short double-stranded RNA molecules, typically 19–25 base pairs in length.^1^ Their antisense strand, which exhibits high sequence specificity, is incorporated into the RNA-induced silencing complex (RISC), while the passenger sense strand is subsequently degraded. The antisense-loaded RISC performs RNA interference (RNAi) by degrading the target mRNA transcript, thereby inhibiting translation and downregulating gene expression.^3^^,^^4^ The miRNA constructs, which are non-protein-coding RNA molecules with approximately 22 nucleotides in length, play crucial roles in modulating biological pathways. Therapeutic miRNA is mainly utilized to suppress target genes expression through sequence-dependent binding to the 3′ untranslated regions (UTRs) of mRNA transcripts. This interaction facilitates mRNA deadenylation and decreases polyA binding protein (PABP) interactions, ultimately leading to translational repression.^1^^,^^5^ The ASOs are single-stranded oligonucleotides, typically ranging from 13 to 25 nucleotides in length, designed to hybridize with complementary RNA transcripts. These targeted molecules facilitate the degradation of their RNA counterparts primarily through the enzymatic action of RNAse H-mediated cleavage.^1^^,^^4^ Compared to traditional small-molecule drugs and protein-based therapies, nucleic acid-based therapeutics offer a more fundamental solution by addressing diseases at the genetic level.

However, the clinical translation of nucleic acid-based therapies remains challenging due to limitations in delivery efficiency, stability, and immune response.^4^^,^^6^^,^^7^ The successful clinical application of nucleic acid therapeutics relies heavily on the development of effective delivery systems, which can protect nucleic acids from degradation, facilitate their cellular uptake, and enhance efficient intracellular trafficking. In recent years, extracellular vesicles (EVs) have attracted considerable interest in preclinical studies.^8^^,^^9^^,^^10^^,^^11^^,^^12^ As natural nanocarriers secreted by virtually all cell types, EVs exhibit excellent biocompatibility, low immunogenicity, and the ability to cross biological barriers, which make them an attractive platform for nucleic acid delivery over conventional viral and synthetic non-viral vectors.^8^^,^^11^^,^^13^^,^^14^

This review provides a comprehensive overview of EV-based strategies for nucleic acid delivery. First, we introduce the major categories of nucleic acid delivery vectors, including EVs, as well as non-EV platforms such as viral vectors and chemically synthesized nanoparticles. We then summarize current EV isolation and purification methods, followed by both classical and emerging techniques for nucleic acid loading into EVs. Finally, we discuss different administration routes for nucleic acid-loaded EVs and their potential clinical applications. By addressing these aspects, we aim to highlight the advantages and challenges of EV-based delivery systems and provide insights for their future optimization in therapeutic applications.

## Current strategies for nucleic acid delivery

Nucleic acids have become an indispensable therapeutic tool for the treatment of a broad spectrum of diseases, including hereditary amyloidogenic transthyretin amyloidosis, cancers, retinal degeneration, hyperlipidemia, orphan diseases, cystic fibrosis, heart disease, diabetes, hemophilia, HIV/AIDS, and so on.^15^^,^^16^^,^^17^^,^^18^^,^^19^^,^^20^^,^^21^^,^^22^^,^^23^^,^^24^^,^^25^^,^^26^^,^^27^^,^^28^^,^^29^^,^^30^^,^^31^ They are particularly suitable for the treatment of diseases with well-characterized genetic causes. One notable example is spinal muscular atrophy (SMA), for which two advanced nucleic acid-based therapies have been developed: nusinersen (Spinraza, Biogen)^32^ and onasemnogene abeparvovec (Zolgensma, Novartis).^33^ Nusinersen is an ASO-dependent therapy that binds to a specific sequence in intron 7 of the survival motor neuron 2 (SMN2) pre-mRNA, correcting aberrant splicing and promoting the production of full-length SMN protein.^32^^,^^34^ This treatment provides clinical benefits for SMA patients across all age groups. In contrast, onasemnogene abeparvovec employs an adeno-associated virus (AAV) vector to deliver a functional SMN1 gene copy, thereby restoring SMN protein levels and improving skeletal muscle function in patients under two years old.^33^ In recent years, nucleic acid-based vaccines have also achieved global acknowledgment, largely due to the success of mRNA vaccines against COVID-19. Spikevax (mRNA-1273 vaccine, Moderna) and Comirnaty (BNT162B2 vaccine, Pfizer BioNTech) have been administered to hundreds of millions worldwide. These mRNA vaccines stimulate host cells to produce the antigenic severe acute respiratory syndrome coronavirus-2 (SARS-CoV-2) spike protein, thereby eliciting protective immunity against the coronavirus disease 2019 (COVID-19).^35^ The pandemic has not only accelerated the development of mRNA vaccines but also propelled nucleic acid-based therapeutics to the forefront of modern medicine.

In theory, a single effective dose of nucleic acids can provide a durable therapeutic effect. However, their clinical application is considerably compromised by several challenges. Nucleic acids exhibit poor stability in the bloodstream because they are rapidly degraded by nucleases and cleared by the mononuclear phagocyte system, leading to short circulation times and inefficient biodistribution to target organs.^4^^,^^7^ Even upon reaching target cells, their internalization is severely restricted due to their negative charge, high molecular weight, and hydrophilicity.^1^^,^^36^ Furthermore, following internalization, only a small fraction (1%–2%) successfully escapes from the endosomes, further diminishing therapeutic efficacy.^37^ Moreover, the activated immune response also poses a challenge for nucleic acid-based therapeutics. For example, Dharmacon has shown that siRNAs can induce interferon responses *in vitro*, potentially leading to cell death. Additionally, other research has indicated that siRNAs containing GU-rich sequences can activate toll-like receptors.^4^ In clinical practice, FDA-approved ASO drugs may be associated with hepatotoxicity, kidney toxicity, and hypersensitivity reactions.^38^ To overcome these barriers, extensive research efforts have been devoted to the development of efficient nucleic acid delivery vectors. These include non-EV delivery systems, such as viral vectors and chemically synthesized nanoparticles (NPs), as well as EVs, which serve as naturally derived carriers.^39^^,^^40^^,^^41^^,^^42^^,^^43^^,^^44^

## Non-EV delivery systems

Viral vectors, such as AAV, lentiviruses, retroviruses, and parvoviruses, have been widely employed in gene delivery due to their high transfection efficiency.^45^^,^^46^^,^^47^^,^^48^^,^^49^^,^^50^^,^^51^^,^^52^ However, their integration into the host genome and their strong immunogenicity frequently lead to inflammation and other adverse effects.^40^^,^^53^ Among these, AAV vectors are most commonly used for clinical applications. Nevertheless, systemic injection of high doses of AAV has been reported to potentially induce severe immune responses, leading to hepatotoxicity, cardiotoxicity, and even death.^53^^,^^54^^,^^55^ Studies have shown that high-dose AAV administration can strongly activate the complement system, causing acute hepatoxicity characterized by elevated liver enzymes levels and liver sinusoidal endothelial cell injury.^55^ The activated adaptive immune responses, accompanied by the activation of cytotoxic T lymphocytes, further exacerbate hepatotoxicity.^55^ As recently reported, Zolgensma triggered subacute liver failure in two pediatric patients. Russia and Kazakhstan even reported two liver failure-associated fatalities following treatment with Zolgensma at 5–6 weeks.^54^ Additionally, the potential chronic cardiotoxicity, characterized by elevated troponin I levels and T-cell-mediated inflammation, as well as acute endothelial injury syndromes, have been observed, highlighting the necessity for stringent dose management and close clinical monitoring.^55^^,^^56^ Additionally, challenges such as rapid clearance by pre-existing antibodies, the generation of neutralizing antibodies, restricted cargo capacity (typically <7 kb), limited genetic capacity, and high production costs further constrain the broader clinical applications of viral vectors.

Chemically synthesized NPs, including lipid-based NPs,^8^^,^^31^ polymeric NPs,^43^ and inorganic NPs (gold NPs, iron oxide NPs, and mesoporous silica NPs)^57^^,^^58^ offer an alternative approach for nucleic acid delivery. Among these, lipid-based NPs such as liposomes and lipid nanoparticles (LNPs) are the most widely used carriers due to their structural stability in physiological fluids and their ability to efficiently encapsulate and deliver nucleic acids. Cationic lipids constitute essential structural components in both liposomes and LNP formulations, significantly enhancing nucleic acid delivery efficacy through several key mechanisms.^31^ First, their positively charged headgroups facilitate electrostatic interactions with negatively charged nucleic acids, thereby improving encapsulation efficiency. Second, the amphiphilic properties of these molecules promote intracellular uptake by destabilizing biological membranes. Third, cationic lipids facilitate endosomal escape through pH-dependent binding with endogenous anionic lipids in endosomes, enabling efficient cytoplasmic release of nucleic acid payloads via membrane disruptions. By optimizing lipid composition, synthesis parameters, and nucleic acid loading strategies, the gene-based therapeutic efficiency of lipid-based NPs has been enhanced, leading to breakthrough gene therapies such as patisiran (Onpattro)^59^ and mRNA-based COVID-19 vaccines.^35^ Lipid-based NPs are favored for their ease of manufacture, modifiability, high nucleic acid loading efficiency, and favorable biocompatibility, all of which have contributed to their clinical success. However, they still face several limitations, including rapid clearance *in vivo*, suboptimal transfection efficiency, and an increased risk of immunogenicity.^60^^,^^61^^,^^62^

Taken together, the limitations associated with viral vectors and chemically synthesized NPs highlight the urgent need for continued development of novel nucleic acid carriers with decreased clearance and toxicity, minimal immunogenicity, improved cargo loading capacity, and improved targeting efficiency.

## Extracellular vesicles

EVs are a heterogeneous group of membrane vesicles secreted by almost all living cells under normal physiological or pathological conditions. EVs used for nucleic acid delivery mainly include exosomes and microvesicles.^63^ Exosomes, with the diameter of 30–150 nm, are generated through a multistep process involving double invagination of the plasma membrane.^64^^,^^65^ The initial invagination of the cell membrane forms a cup-shaped structure known as the early-sorting endosome (ESE). ESEs give rise to late-sorting endosomes, which undergo the second invagination of plasma membrane, leading to the formation of multivesicular bodies (MVBs) containing intraluminal vesicles (future exosomes). The MVBs can either fuse with lysosomes or autophagosomes for degradation, or alternatively, fuse with the cell membrane to release exosomes into the extracellular space.^64^^,^^65^ Microvesicles, with a larger diameter of 100–1,000 nm, are generated directly through outward budding or shedding of the plasma membrane.^13^ EVs facilitate intercellular communication by transferring biological components including proteins, lipids, and nucleic acids from parental cells to nearby or distant recipient cells. Their nanoscale structure and natural ability to transport bioactive molecules have made them a promising drug delivery platform. In preclinical studies, EVs have been extensively investigated as carriers for delivering chemotherapy drugs, protein therapeutics, and nucleic acid-based therapies across a variety of disease models.^66^^,^^67^^,^^68^^,^^69^^,^^70^ Owing to their natural biocompatibility, low immunogenicity, and ability to cross biological barriers, EVs have emerged as a promising platform for targeted drug and nucleic acid delivery in therapeutic applications.

## Preparation methods of EVs

**Figure 1:**
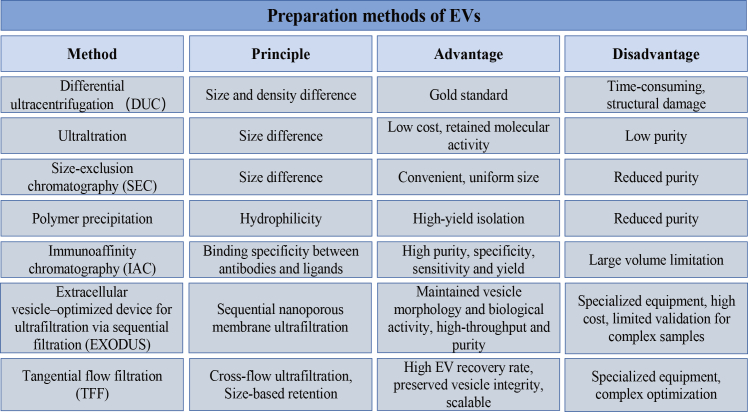
Preparation methods of extracellular vesicles and their respective principles, advantages, and disadvantages

Given the increasing interest in employing EVs as nucleic acid delivery systems, establishing reliable and scalable preparation procedures is crucial for ensuring consistency and functional performance. To support downstream engineering and therapeutic applications, various EV isolation strategies have been developed according to differences in density, size, shape, and surface membrane proteins. The most commonly used techniques include differential ultracentrifugation (DUC), ultrafiltration, size-exclusion chromatography (SEC), polymer precipitation, immunoaffinity chromatography (IAC), and commercially available kits such as exoEasy Maxi Kit (QIAGEN), MagCapture Exosome Isolation Kit PS (Wako), and Minute Hi-Efficiency Exosome Precipitation Reagent (Invent), along with recently introduced innovative platforms such as the nanofluidics-based EXODUS (extracellular vesicle-optimized device for ultrafiltration via sequential filtration) system and tangential flow filtration (TFF) (Figure 1).^71^^,^^72^^,^^73^^,^^74^^,^^75^^,^^76^^,^^77^

## Differential ultracentrifugation

DUC is considered as the gold standard for EV isolation and is widely used owing to its capacity to process both small and large fluid volumes. This method separates EVs based on size and density differences in biological samples, yielding relatively high concentrations of EVs. However, DUC requires specialized, costly equipment, is time-consuming, and exposes EVs to shear forces that may cause structural damage, lipoprotein co-isolation, and aggregation, thereby complicating downstream analyses.^42^

## Ultrafiltration

Ultrafiltration is another size-based isolation technique that utilizes membranes with different molecular weight cut-offs to selectively separate EVs. Although this method preserves the biological activity of EVs, it also exhibits obvious limitations. Ultrafiltration frequently results in low recovery rates and limited purity due to vesicle clogging and the non-specific retention of contaminants with sizes comparable to EVs.^73^

## Size-exclusion chromatography

SEC separates EVs based on size, using porous gel filtration columns. Larger macromolecules are excluded from the gel pores and eluted earlier, while smaller molecules are retained and eluted later.^74^ SEC is convenient, cost effective, and preserves EV structural integrity and biological activity. Commercial SEC-based kits, such as qEV separation columns, EVSecond purification columns, and Exo-spin exosome purification columns, are available for EV isolation.^78^ However, particles with similar size may also be retained, reducing the overall purity.^74^

## Polymer precipitation

Polymer precipitation, analogous to the principle of ethanol-mediated nucleic acid precipitation, employs polyethylene glycol (PEG; molecular weight 6,000–20,000 Da) to create a hydrophobic microenvironment around EVs, thereby inducing their precipitation. This method is easily scalable and enables high-yield production. However, PEG also precipitates other water-soluble molecules, such as nucleic acids and lipoproteins, which compromise purity of the isolated EVs.^71^^,^^72^

## Immunoaffinity chromatography

IAC isolates EVs based on specific antibody-ligand interactions. Common EV surface markers, including CD63, CD9, CD82, Annexin, and Alix, serve as targets for EV capture.^71^ Several commercial IAC kits such as Exosome-human CD63 Isolation Reagent (Thermo Fisher) and Exosome Isolation Kit CD81/CD63 (Miltenyi Biotec) are available for high-specificity isolation.^73^ IAC offers high specificity, sensitivity, purity, and yield, which make it particularly suitable for small-volume samples. However, the high cost of antibodies limits its application for large-scale sample processing.

## Other innovative technologies

In addition to the conventionally used methods mentioned above, several emerging platforms have been introduced to improve the scalability, throughput, and reproducibility of EV preparation.

EXODUS is a recently reported nanofluidics-enabled platform that integrates sequential nanoporous membrane filtration under dynamically regulated pressure and laminar flow to enrich EVs within approximately 30–200 nm range, while simultaneously reducing membrane clogging and excessive shear stress.^75^ Through the combination of multi-stage, size-selective filtration and automated pressure control, EXODUS is designed to enhance the reproducibility of EV preparation across various sample types and processing volumes and to support workflows that demand standardized inputs for downstream analyses. The original report indicates that EXODUS can achieve higher recovery rates, faster processing times, and lower protein contamination than conventional ultracentrifugation, which highlight its potential for scalable and high-throughput EV isolation.^75^ Nevertheless, the broader adoption may be restricted by the need for specialized instrumentation and careful pressure calibration, and its performance in highly viscous or particulate-rich biofluids (e.g., ascites or lipoaspirate) still requires systematic validation.^75^

TFF is another scalable size-based approach that concentrates EVs using cross-flow ultrafiltration.^76^^,^^77^ In this process, the sample stream runs parallel to a semi-permeable membrane, allowing the solvent and small solutes to pass through while retaining vesicles in the retentate. Compared with dead-end filtration, TFF mitigates membrane fouling and facilitates the removal of soluble protein contaminants while maintaining EV size distribution, morphology, and marker expression.^76^^,^^77^ Importantly, it is readily adaptable to large-volume and clinical-grade processing. However, method performance depends on operational parameters (e.g., membrane molecular weight cut-off, transmembrane pressure, and shear rate). Residual serum proteins or preferential enrichment of specific EV subpopulations may still occur, which should be considered when interpreting downstream functional readouts.

Each isolation strategy imposes distinct physical and biochemical stresses on EVs. The selection of preparation method can influence downstream nucleic acid loading and delivery outcomes. For example, prolonged processing and high g-forces in some workflows may alter EV integrity and surface composition, which could, in turn, affect electroporation efficiency, fusion-based loading, and ligand-directed targeting. Conversely, gentler and scalable workflows may better preserve membrane and protein features relevant to reproducible cargo encapsulation. Therefore, the EV isolation method should be selected in concert with the intended loading strategy (endogenous vs. exogenous), cargo stability requirements, dosing consistency, and translational scalability.

## Methods of nucleic acid loading into EVs

**Figure 2:**
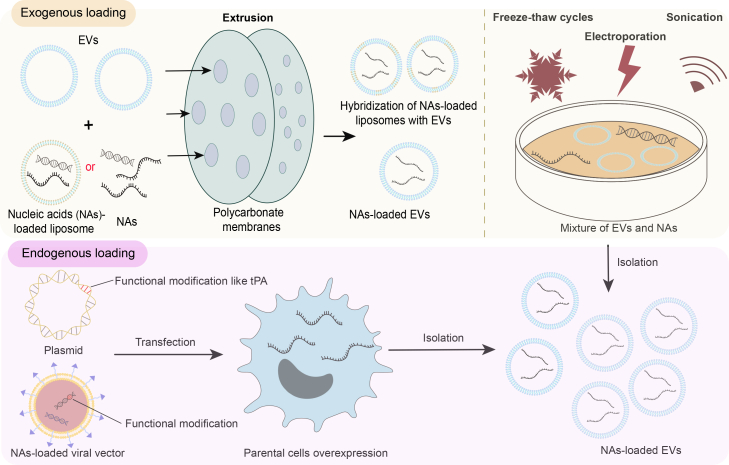
The extracellular vesicle loading methods for drug delivery, including endogenous loading methods and exogenous loading methods such as extrusion, freeze-thaw cycles, electroporation, and sonication

The natural targeting capabilities, immune inertia, high biocompatibility, and intrinsic role in intercellular RNA transport of EVs make them attractive platforms for nucleic acid-based therapies. Broadly, nucleic acids can be incorporated into EVs through endogenous loading (via parental cell engineering during EV biogenesis) or exogenous loading (direct manipulation of isolated EVs) (Figure 2).

## Endogenous loading

Endogenous loading involves the genetic engineering of parental cells to increase the intracellular concentration of the nucleic acid of interest, which are subsequently packaged into EVs during biogenesis.^79^^,^^80^ This approach is particularly suitable for RNAs and proteins that cannot be directly loaded into EVs. For example, Zhang et al. engineered mesenchymal stem cell (MSC)-derived EVs to overexpress circCDK13, miR-141-3p, or miR-17-5p (termed circCDK13^OE^-EVs, miR-141-3p^OE^-EVs, or sEVs^17−OE^, respectively) by transfecting parental cells with lentiviral vectors to facilitate diabetic wound healing and hypertrophic scar treatment.^81^^,^^82^^,^^83^ Further RT-qPCR detection revealed that genes overexpressed in parental cells were successfully incorporated into secreted EVs. Compared with the vector group-derived EVs, the expressions of circCDK13, miR-141-3p, and miR-17-5p in EVs increased about 200-fold, 17-fold and 15-fold, respectively. Interestingly, the expression of circCDK13 and miR-141-3p in parental cells (100-fold and 170-fold, respectively) did not completely align with their enrichment in EVs (200-fold and 17-fold, respectively). This discrepancy might be related to the covalent closed-loop structure of circCDK13, which conferred it enhanced stability and resistance to RNase degradation.^81^^,^^82^^,^^83^ In particular, circCDK13^OE^-EVs interacted with insulin-like growth factor 2 mRNA binding protein 3 (IGF2BP3) in an m6A-dependent manner, enhancing the mRNA stability and protein levels of c-MYC and CD44, thereby promoting dermal fibroblast proliferation and keratinocyte migration to accelerate diabetic wound healing.^81^

More complex genetic engineering approaches have also been developed. Li et al. exploited CasRx-gRNA-loaded EVs to achieve rapid and transient inhibition of target gene expression for acute disease treatment.^84^ To improve the packaging efficiency of CasRx, three short signal peptides, namely tPA, the mouse Ig heavy chain, and human insulin, which are required for protein secretion, were integrated into the CasRx-gRNA plasmid system. Then, 293T cells were transfected with these engineered plasmids, and their EVs were collected by ultracentrifugation. As a result, the tPA-fused group exhibited the highest CasRx loading in EVs, achieving an ∼8-fold increase compared to the constructs lacking signal peptides.^84^ Besides short signal peptides, additional elements such as post-translational modifications (e.g., N-myristoylation), cell-penetrating peptides (e.g., PF14), and EV-enriched surface proteins (e.g., CD63, CD9, milk fat globule epidermal growth factor VIII, prostaglandin F2 receptor negative regulator, and BASP1) have also been employed to improve EV cargo loading efficiency.^85^^,^^86^^,^^87^^,^^88^^,^^89^^,^^90^

Numerous systems have been developed for the delivery of small RNAs. However, the efficient enrichment of long mRNAs in EVs remains a major challenge. To address this problem, Gu et al. constructed an innovative approach that utilized a retrovirus-like capsid protein (Arc) and its 5′ UTR to improve mRNA packaging into leukocyte-derived EVs.^91^ Concretely, primary bone marrow-derived leukocytes were transfected with a DNA vector encoding the cargo mRNA, Arc capsid protein, and Arc 5′ UTR (A5U). The Arc protein self-assembled into virus-like capsids, facilitating the encapsulation of the cargo mRNA, which was subsequently secreted within EVs and transferred into recipient neurons. Additionally, the A5U element stabilized the capsid, further increasing both mRNA packaging efficiency and transduction efficiency. The GFP fluorescence-labelled mRNA transcripts were employed to explore the mRNA loading efficiency, and the fluorescence intensity of the purified EVs was boosted by ∼6-fold in the presence of Arc and A5U, indicating the significantly promoted mRNA cargo encapsulation capability.^91^ Furthermore, these EVs retained leukocyte-derived endothelial adhesion molecules, enabling blood-brain barrier (BBB) penetration and targeted delivery to neuroinflammatory regions, making them a promising tool for central nervous system (CNS) therapies.^91^ Similarly, another capsid protein homolog, the mammalian retrovirus-like protein PEG10, has been repurposed to pseudotype virus-like particles for nucleic acid packaging and delivery.^92^

Endogenous loading also includes non-genetic strategies. Yang et al. developed a cellular nanoporation (CNP) system. In this system, cells cultured on a nanochannel array with a 500-nm pore size were exposed to controlled electrical pulses. This process facilitated the transfer of nucleic acids from the buffer into cells.^93^ This method induced local heat shock responses, activating the p53-TSAP6 signaling pathway and significantly increasing EV secretion. Furthermore, local membrane injuries elevated the intracellular calcium levels, promoting a 2-fold increase in MVBs and an 8-fold increase in intraluminal vesicles. Ultimately, it boosted EV production by over 50-fold and mRNA transcript loading by more than 1,000-fold.^93^

## Exogenous loading

Exogenous loading directly introduces nucleic acids into pre-isolated EVs through physical or chemical methods, including electroporation, sonication, freeze-thaw cycling, extrusion, and commercial transfection kits.^78^^,^^94^

Electroporation is one of the most widely used techniques. It applies short, high-voltage pulses to EVs and nucleic acids, creating transient pores in the EV membrane that facilitate nucleic acid entry.^78^ This method is simple and time efficient, and it has been applied to load both nucleic acids and small-molecule drugs. For instance, Shamshiripour et al. collected peripheral blood mononuclear cell-derived EVs by using SEC and loaded them with both the hydrophilic chemotherapy drug doxorubicin hydrochloride and VEGF-A siRNA to inhibit glioma growth by reducing angiogenesis.^95^ Doxorubicin hydrochloride was loaded through incubation at room temperature for 30 min, while VEGF-A siRNA was introduced into the EVs via electroporation at 400 V and 125 μF. The loading efficacy, determined by spectrofluorimetry, was 25.17% ± 2.69% in exosomes and 32.01% ± 2.98% in microvesicles. Following electroporation, exosomes maintained an ellipsoid or spherical morphology, though ∼35% aggregation was observed. Nevertheless, the microvesicles showed pronounced aggregation under identical conditions, which was alleviated when the voltage was reduced to 200 V, albeit with a slight increase in microvesicle size.^95^ Apart from EV aggregation, electroporation is associated with several other drawbacks, including membrane disruption, potential content leakage, and RNA precipitation. Thus, protocol optimization is critical to maximize loading efficiency while minimizing adverse effects.

Sonication employs low-frequency ultrasound waves to temporarily disrupt the EV membrane, enabling the incorporation of small molecules and protein cargo, especially hydrophobic drugs.^78^ This technique has been shown to alter membrane rigidity, facilitating structural reassembly and cargo loading. Lamichhane et al. described sonication as an optimized protocol to load small RNAs into EVs.^96^ They demonstrated that sonication at 35 kHz for up to 180 s did not significantly alter EV size and number, suggesting minimal EV agglomeration. However, prolonged sonication (more than 30 s) led to nucleic acid degradation. But, aggregation induced by sonication at 35 kHz for 30 s was ∼12-fold less than that induced by electroporation at 400 V, 125 μF with two pulses.^96^ Since then, sonication has been used to load small RNAs into EVs. For example, bone marrow-derived MSC-EVs loaded with miR-138-5p via sonication under the conditions of 4°C, 60 W power, 6 cycles of 3 s pulses and 10 s pauses resulted in a 9,000-fold increase in miR-138-5p expression.^97^ These miR-138-5p-loaded EVs effectively inhibited TGF-β signaling pathway activation and collagen synthesis, demonstrating potential in pancreatic cancer therapy.^97^ Although sonication provides a relatively effective loading efficiency for small RNAs, the shear stress and heat generated during the sonication process may damage the plasma membrane structure of EVs and the activity of small RNAs. Optimizing the sonication conditions is feasible to ensure the loading efficiency while reducing the damage to EVs and small RNAs.

The freeze-thaw cycling method involves repeatedly freezing EV-cargo mixtures at −80°C or in liquid nitrogen, followed by thawing at room temperature.^98^ Although convenient and cost effective, freeze-thaw cycling is associated with a low encapsulation efficiency, reduced EV stability, increased aggregation, and potential size alterations.

Extrusion forces EVs through a membrane filter under high pressure, disrupting and reconstructing the membrane to encapsulate therapeutic agents.^98^ This technique achieves high drug-loading efficiency but compromises the natural structure and biological activity of EVs. Despite these drawbacks, extrusion is valued for its scalability and effectiveness in loading large quantities of therapeutic agents.

In addition to these methods, several other strategies have been developed to improve nucleic acid loading efficiency. Techniques such as transmembrane pH gradients, heat shock, and hybridization with other nanocarriers (e.g., liposomes) have been used to disrupt EV membrane structures and enhance cargo encapsulation.^99^^,^^100^^,^^101^ A novel hybridization-based strategy was used to improve the encapsulation efficiency of siRNAs. Based on the theory put forward by Kooijmans et al., siRNA loading occurs through a volume exchange between the EV lumen and the siRNA-containing extravesicular medium, driven by a concentration gradient.^102^^,^^103^ Therefore, the concentration of siRNAs in the extravesicular medium and the ratio of medium inside to outside the EVs are the two main parameters that influence the loading efficiency. To optimize these parameters, researchers pre-accumulated the siRNA of interest into oligomer-stabilized calcium phosphate NPs (CaP-NPs) via precipitation and concentrated EVs through a dehydration-rehydration process. The concentrated EVs and siRNA-loaded CaP-NPs were then mixed, and siRNA was loaded into EVs via dual asymmetric centrifugation, which significantly improved the encapsulation efficiency, achieving 51.8% ± 2.5%.^103^ Another innovative method involves spontaneous hybridization with lipid crystalline nanoparticles (LCNPs). Bader et al. developed a hybrid EV-LCNP system (HEVs) to improve nucleic acid delivery efficiency while preserving EV bioactivity.^104^ LCNPs were synthesized by combining a lipid/surfactant-containing organic phase with a nucleic acid-containing aqueous phase via microfluidic mixing. The LCNPs and MSC-derived EVs were then mixed in a 1:1 particle ratio under pH 7.4. This pH-induced structural transformation, from an inverse hexagonal (H_II_) phase to a non-lamellar phase, facilitated controlled hybridization with EVs, leading to enhanced target inhibition and expression efficiencies, while maintaining the biological activity of EV membrane proteins.^104^

Commercial transfection reagents, such as HiPerFect, Lipofectamine 2000, and the Exo-Fect Exosome Transfection Kit, are also employed for nucleic acid loading into EVs. These kits offer straightforward protocols for loading various nucleic acids, including siRNAs, gRNAs, and gRNA-Cas9 complexes, directly into EVs.

In summary, endogenous nucleic acid loading into EVs allows precise control over cargo type and content, as well as preserving EV bioactivity. The exogenous loading methods for nucleic acids into EVs offer various advantages, including ease of use and scalability. However, they often face challenges related to EV integrity and loading efficiency. Emerging techniques, such as CNP and LCNP-EV hybridization, show promise in enhancing nucleic acid encapsulation while maintaining EV bioactivity. The choice of loading method should be guided by the properties of the therapeutic agent, desired loading efficiency, EV membrane structure, and subsequent experimental requirements (Table 1).

**Table 1:** Comparison of methods for loading nucleic acids into extracellular vesicles

Method	Strategy	Cargo	Integrity of EVs	Loading efficiency	Reference
Endogenous loading	lentivirus transfection	circRNAs	preserving structural integrity	>200-fold increase relative to circRNA^NC^-EVs (mock transfection)	Huang. et al.^81^
	lentivirus transfection	miRNAs	preserving structural integrity	>10-fold increase relative to miRNA^NC^-EVs	Meng. et al.^82^, Wei. et al.^83^
	integration of short signal peptides (tPA)	CasRx-gRNA	preserving structural integrity	8-fold increase relative to the group without signal peptide modification	Li. et al.^84^
	A5U	mRNAs	preserving structural integrity	6-fold increase relative to the group without Arc 5′ UTR	Gu. et al.^91^
	cellular nanoporation	mRNAs	boosting EV production by over 50-fold compared to conventional bulk electroporation (BEP)	>1, 000-fold increase relative to BEP or lipofectamine 2000 groups	Yang. et al.^93^
Exogenous loading	electroporation	siRNAs	35% aggregation	25.17% ± 2.69%	Shamshiripour. et al.^95^
	sonication	small RNAs	aggregation, less than electroporation	9,000-fold increase relative to the EV-negative control	Zhou. et al.^97^
	freeze-thaw cycling	siRNAs	reduced stability	about 0.43 fmol/10^11^ EVs	Roerig. et al.^103^
	extrusion	siRNAs	disrupting and reconstructing	about 0.25 fmol/10^11^ EVs	Roerig. et al.^103^
Emerging loading	DAC/calcium phosphate-NPs	siRNAs	N.A.	about 25 pmol/10^11^ EVs	Roerig. et al.^103^
	hybrid EV-lipid crystalline nanoparticle system (HEVs)	siRNAs	preserving membrane protein and enzyme activity	about 30%–40% of HEVs contained siRNA	Bader. et al.^104^

Importantly, nucleic acid loading strategies not only determine the encapsulation efficiency but also influence key surface characteristics of EV-cargo complexes, including membrane integrity and protein composition. These properties directly affect how nucleic acid-loaded EVs interact with biological barriers following administration, such as systemic clearance, immune recognition, tissue penetration, and endosomal escape. Therefore, loading strategies and administration routes should be considered in a coordinated manner rather than independently.

## Administration routes of nucleic acid-loaded EVs

**Figure 3:**
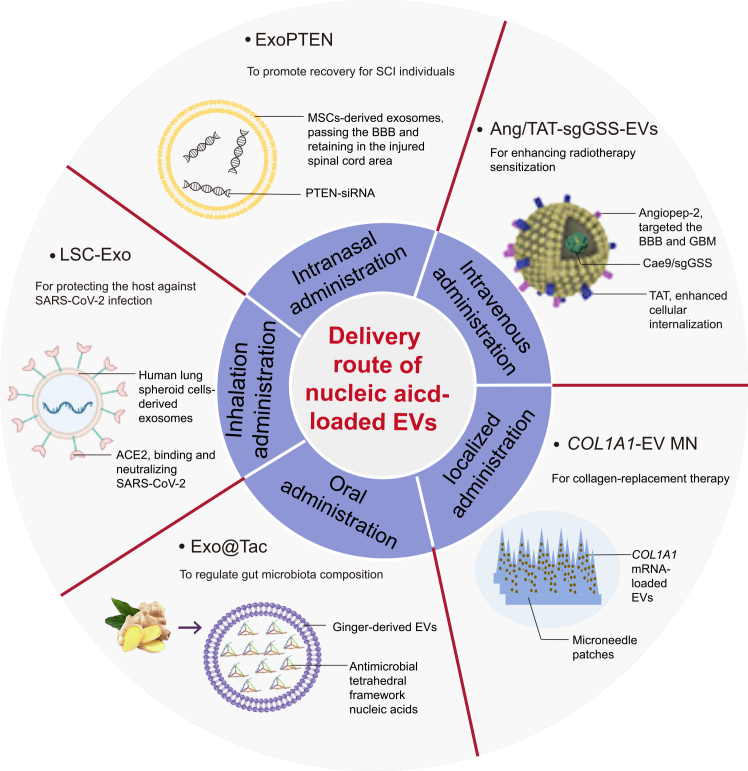
Administration routes of nucleic acid-loaded extracellular vesicles, including intravenous, local, oral, intranasal, and inhalation delivery

The primary administration routes of nucleic acid-loaded EVs mainly include intravenous, localized, oral, intranasal, and inhalation delivery (Figure 3 and Table 2). Different administration routes lead to different biodistribution, pharmacokinetics,^10^ and therapeutic outcomes of nucleic acid-loaded EVs, which are influenced by biological barriers, cargo properties, EV sources, and EV surface characteristics.

**Table 2:** Comparison of different administration routes of nucleic acid-loaded EVs

Administration routes	Highlights	Advantages	Limitations	Design implications	Distribution of EVs	Applications	Reference
Intravenous injection	the predominant route for delivering EVs	no absorption barrier, and precise control of systemic exposure/dose	systemic clearance and off-target distribution	minimize RES uptake and add tissue/cell tropism modification	predominant distribution in the liver, lung, and spleen	Tumors and various other diseases	Su. et al.^10^, Liu. et al.^107^, Kamerkar. et al.^112^, Fu. et al.^113^
Localized administration	delivering EVs through gelatin or microneedle patch	high local exposure, reduced systemic burden, and sustained release	poor stability, poor deep penetration, storage problem, and difficulty in controlling release	select a depot format, tune release kinetics and retention, and promote tissue penetration	mainly enriched at target sites	SCI, skin regeneration, wound healing, anti-aging, etc.	Fang. et al.^115^, Yao. et al.^116^, You. et al.^117^
Oral administration	plant- and milk-derived EVs	non-invasiveness, ease of use, and high patient compliance	mucosal barriers, enzyme degradation, and strong acidic conditions in the gastrointestinal tract, diminishing the bioavailability	prioritize intrinsically robust EV sources, design for intestinal uptake, and favor degradation-resistant cargos	exhibiting a relatively uniform distribution across multiple organs, and superior intestinal distribution	Inflammatory bowel disease, aortic dissection, PD, etc.	Su. et al.^10^, Pomatto. et al.^118^, Umezu. et al.^119^, Zhang. et al.^120^, Liu. et al.^121^
Intranasal administration	non-invasive route for direct brain and lung delivery	non-invasiveness and bypasses hepatic/intestinal metabolism	mucociliary clearance and enzymatic degradation	increase nasal residence time, protect cargo in nasal environment, and tune EV surface/size for epithelial transport and target cell uptake	distributing in the brain within 30 min, and primarily accumulating in the lungs at 24 h post-intranasal administration	AD, SCI repair, etc.	Zhuang. et al.^124^, Peng. et al.^125^, Guo. et al.^126^
Inhalation administration	achieving direct pulmonary drug delivery via nebulizers	non-invasiveness and high lung deposition	compromising the integrity and difficult to control the dosage	validate aerosolization stability, control deposition, and dosing reproducibility, mitigate airway barriers	predominant distribution in the lung at 2 h post-inhalation administration	SARS-CoV-2 infection, lung tumors, etc.	Popowski. et al.^127^, Wang. et al.^128^, Liu. et al.^129^

EV, extracellular vesicles; RES, reticuloendothelial system; AD, Alzheimer disease; PD, Parkinson disease; SCI, spinal cord injury.

Advantages describe route-intrinsic benefits, whereas design implications highlight route-specific formulation/engineering actions aligned with dominant *in vivo* barriers.

Different administration routes expose nucleic acid-loaded EVs to distinct biological filters, resulting in divergent biodistribution and clearance profiles. For example, intravenous delivery often undergoes rapid clearance by the reticuloendothelial system, with preferential accumulation in the liver and spleen,^10^ whereas intranasal administration enhances brain accessibility by engaging olfactory/trigeminal pathways.^10^^,^^105^ In some settings, this delivery route enriches EV signals in the lungs while reducing hepatic predominance.^105^ Local administration bypasses systemic clearance, improving local exposure and residence time at the lesion site. Importantly, these route-dependent distribution and clearance profiles determine which barriers are the most rate limiting (e.g., extracellular nuclease exposure, immune sensing, tissue penetration, and endosomal escape). Therefore, route selection is most informative when considered together with (1) cargo modality and intracellular site-of-action, (2) nucleotide chemistry that affects stability and immunogenicity, and (3) EV surface features that control tissue/cell interactions.

First, cargo modality dictates intracellular destination requirements. Small cargoes (siRNAs and many ASO modalities) primarily act in the cytoplasm and can be adequately delivered through systemic routes with moderate targeting.^1^ Larger or unstable cargoes, such as mRNAs, require both protection from nuclease degradation and efficient endosomal escape, which favors routes that prolong circulation and enhance targeting. However, DNA cargoes and some genome-editing systems require additional nuclear access steps, increasing dependence on efficient uptake and intracellular trafficking.

Second, nucleotide chemistry can expand feasible routes by improving stability and reducing immune activation. Clinically established oligonucleotide chemistries (e.g., phosphorothioate backbones; 2′-O-methyl/2′-O-methoxyethyl/2′-fluoro sugar modifications) can increase resistance to degradation and support systemic exposure.^3^ In contrast, mRNA benefits from base modifications (e.g., pseudouridine-class substitutions) that mitigate immunogenicity and enhance translation, which is crucial for routes that expose cargo to immune surveillance or involve prolonged circulation.^1^

Third, EV engineering features (origin, surface ligands, stealth coatings, and microenvironment-responsive designs) should be considered route synergistic.^106^ Systemic routes require strategies to reduce non-target uptake, while local or mucosal routes focus on overcoming extracellular barriers and ensuring prolonged release at the target site.^10^ Importantly, the choice of delivery route is often tailored to the disease type, as different diseases may require more specific tissue targeting or longer treatment durations.

This comprehensive “route-cargo-EV engineering” approach guides the optimal delivery system for each therapeutic goal.

## Intravenous administration

Intravenous administration is the most widely used route for drug delivery. However, it often results in low bioavailability due to systemic clearance and off-target distribution. Optimizing EV targeting strategies may improve therapeutic efficacy and enhance clinical translation. Encapsulating nucleic acids in EVs offers protection from nucleases in the bloodstream, thereby increasing stability. Additionally, surface modifications with short peptides are frequently employed to improve tissue and cellular targeting. For instance, Liu et al. developed angiopep-2 (Ang) and *trans*-activator of transcription (TAT) peptide-co-expressing HEK 293T cells to enhance EV targeting.^107^ Ang binds to low-density lipoprotein receptor-related protein 1 (LRP1), facilitating BBB penetration, whereas TAT, a potent cell-penetrating peptide, enhances cellular internalization and nuclear entry. After collecting Ang/TAT-modified EVs via centrifugation, the Cas9 protein-sgRNA complex was loaded into EVs via electroporation, generating Ang/TAT-sgGSS-EVs.^107^ Following intravenous administration, these EVs efficiently targeted glioblastoma tissue, with tumor cells exhibiting a high glutathione synthetase (GSS) gene-editing efficiency (∼67.2%) and minimal off-target effects. GSS inhibition disrupted glutathione synthesis, leading to glutathione peroxidase 4 (GPX4) inactivation and iron accumulation, ultimately inducing ferroptosis and enhancing radiotherapy sensitization.^107^

Several other short peptides such as TBP-CP05,^108^ M2pep,^109^ NP41,^110^ and P-selectin binding peptide (PBP)^111^ have also been used to improve EV targeting. TBP-CP05, a bi-functional peptide, binds specifically to CD63 on EVs via CP05, while its TBP domain targets pre-osteoclasts and osteoclasts by binding to tartrate-resistant acid phosphatase.^108^ Additionally, M2pep, NP41, and PBP have been shown to specifically bind to M2-like tumor-associated macrophages (TAMs), nerve cells, and injured kidney, respectively.^109^^,^^110^^,^^111^ In order to enhance clinical applicability, Kamerkar et al. developed a simple and effective EV-based platform for delivering signal transducer and activator of transcription 6 (STAT6) ASO (termed exoASO-STAT6) for monotherapy anticancer treatment.^112^ Stringently purified HEK 293 cell-derived EVs exhibited high expression of PTGFRN and strong affinity for myeloid cells, including TAMs. These EVs were surface loaded with STAT6 ASO, leading to repolarization of TAMs from a pro-tumor M2-like phenotype to an anti-tumor M1-like phenotype. After intravenous injection, exoASO primarily distributed in the liver, where it was internalized by myeloid cells, leading to tumor microenvironment remodeling and significant inhibition of tumor growth in hepatocellular carcinoma models.^112^ To further evaluate gene therapy efficiency *in vivo*, Fu et al. designed a graphene quantum dot (GQD)-based EV system to visualize the hybridization process between miRNAs and mRNAs.^113^ In this system, GQDs and Cy5-tagged miR-193a-3p formed a complex through π–π stacking interactions. While bound to GQDs, the fluorescence signal of miRNAs was quenched. Upon cellular uptake, miRNA escaped from endosomes and hybridized with the target mRNA, restoring fluorescence and allowing real-time tracking of miRNA activity in living cells.^113^

## Localized administration

To enhance EV accumulation and ensure sustained release at target sites, localized administration strategies have been developed. These methods improve therapeutic efficacy while minimizing systemic clearance.

For example, ASO-loaded EVs were administered intracerebroventricularly to reduce α-synuclein aggregation and restore locomotor function in a transgenic Parkinson disease (PD) mouse model.^114^ Similarly, a gelatin methacryloyl (GelMA)-integrated microneedle array was designed to encapsulate MSCs, enabling the sustained release of EVs for spinal cord injury (SCI) treatment.^115^ Gelatin-based and microneedle-assisted delivery systems have also been widely explored for skin regeneration. For instance, a hydrogel microneedle patch (MNP) loaded with mitochondria-rich EVs derived from metformin-treated, adipose-derived stem cells significantly accelerated chronic wound healing.^116^ Considering that miR-17-5p overexpression protects endothelial cells from high glucose-induced injury and promotes diabetic wound healing, EVs^17−OE^ (described earlier) were encapsulated in a GelMA hydrogel to serve as a bioactive wound dressing.^83^ Upon administration, GelMA facilitated the sustained and stable release of EVs^17−OE^ at the wound sites for approximately 20 days, significantly inhibiting p21 (a cell cycle inhibitor), reversing cell senescence, stimulating proliferation, enhancing angiogenesis and collagen deposition, and effectively promoting diabetic wound healing *in vivo*.^83^

In another application, You et al. developed a hyaluronic acid (HA) microneedle patch for intradermal delivery of extracellular matrix α1 type-I collagen (*COL1A1*) mRNA-loaded EVs to facilitate collagen replacement in photoaged skin.^117^ Using the CNP technique, *COL1A1* plasmid DNA was transfected into neonatal human dermal fibroblasts, generating *COL1A1* mRNA-enriched EVs (*COL1A1*-EVs). These EVs were mixed with a 15% HA solution to form microneedle patches (*COL1A1*-EV MN) with a base diameter of 400 μm and a height of 1,000 μm. When applied to the dorsal skin of mice, *COL1A1*-EV MN penetrated the epidermis and reached the dermis (516 ± 76 μm), dissolving completely within 15 min. This enabled efficient distribution of EVs in the dermis and subcutis, resulting in long-lasting collagen protein replacement and a significant reduction in wrinkle length and depth, which persisted for up to two months.^117^

Gelatin- and microneedle-based nucleic acid-loaded EV delivery strategies show great potential for treating skin-related diseases due to their minimally invasive nature and ease of application. However, other localized administration routes, such as intracerebroventricular, intrahepatic, or intraovarian injections, are highly invasive, require specialized surgical expertise, and are not suitable for long-term treatment, limiting their clinical feasibility.

## Oral administration

Oral administration is a non-invasive, convenient, patient-friendly, and widely accessible drug delivery method. However, most synthetic NPs exhibit poor oral bioavailability due to their low stability in the digestive tract and inefficient uptake by intestinal epithelial cells. In contrast, food-derived EVs have emerged as a promising solution, offering advantages such as edibility, biocompatibility, and efficient gastrointestinal absorption.

For example, *Citrus sinensis*-derived EVs have been successfully used for the oral delivery of mRNA vaccines.^118^ MiRNA-loaded acerola juice-derived EVs achieved target gene inhibition in the liver and small intestine, with peak effects observed 24 h post-gavage.^119^ Similarly, anti-tumor necrosis factor alpha (TNF-α) siRNA-loaded bovine milk-derived EVs effectively reduced inflammation in a rat model of inflammatory bowel disease.^120^ Additionally, EVs derived from green tea, which encapsulated the antagomir-targeting cardiac apoptosis-associated piwi-interacting RNAs (piRNAs), have shown potential in reducing the incidence of aortic dissection.^121^ This is achieved by downregulating myocyte enhancer factor 2D (MEF2D) and matrix metallopeptidase 9 (MMP9), inhibiting the phenotypic transformation of aortic smooth muscle cells.^121^ Interestingly, Cui et al. developed a ginger-derived EV system loaded with antimicrobial peptide (AMP)-modified antimicrobial tetrahedral framework nucleic acids (tFNAs) for PD treatment.^122^ EVs were isolated via ultracentrifugation, and self-assembling tFNAs were loaded by electroporation to yield Exo@tac, which exhibited superior acid resistance and prolonged gastrointestinal retention. Following oral administration, Exo@tac normalized the composition of gut microbiota, influenced microbiota-gut-brain axis-related macrophages, neuromicroglia, and intestinal endocrine cells, reduced inflammation and apoptosis in the CNS, enhanced the production of serotonin (5-HT) and dopamine precursors, decreased α-synuclein accumulation, and improved both motor symptoms and pathological features in a PD mouse model.^122^

Oral administration offers multiple advantages as a non-invasive and effective drug delivery route. However, its application is currently limited to food-derived EVs, which exhibit natural stability and compatibility with the gastrointestinal tract, making them suitable for future therapeutic development.

## Intranasal administration

A substantial body of research highlights the potential of intranasal administration as a non-invasive route for direct brain delivery, facilitating superior accumulation and prolonged retention at injury sites.^123^ Intranasal drug delivery enables drug transport across a single epithelial layer into systemic circulation or bypasses the BBB via olfactory and trigeminal nerves for direct brain entry.^10^ Zhuang et al. found that EVs could be detected in the brain tissue within 30 min of intranasal administration, indicating rapid translocation of EVs to the brain.^10^^,^^124^ Notably, intranasally administrated EVs exhibited superior brain accumulation at both 1 h and 24 h compared to intravenously administered EVs.^123^ These advantages—rapid onset, non-invasiveness, and the ability to bypass hepatic/intestinal metabolism—make intranasal delivery a promising administration route for treating CNS diseases.^10^

Increased miR-206-3p expression in the plasma and temporal cortex is a hallmark of AD pathology.^125^ To counteract this, Peng et al. engineered MSC-derived EVs loaded with miR-206-3p antagomir (MSC-EVs-anta) via electroporation to inhibit miR-206-3p function. Following intranasal administration, brain-derived neurotrophic factor (BDNF) expression was upregulated, promoting hippocampal neurogenesis and synaptic plasticity, reducing Aβ deposition, and alleviating cognitive deficits. This treatment significantly improved learning and memory functions in AD mouse models, demonstrating promising therapeutic potential.^125^ For SCI repair, Guo et al. developed MSC-derived EVs loaded with siRNA targeting phosphatase and tensin homolog (ExoPTEN).^126^ PTEN is widely expressed in neurons and regenerating axons, acting as a key intrinsic inhibitor of corticospinal neuron regeneration by suppressing mammalian target of rapamycin (mTOR) activity. Following intranasal administration, ExoPTEN successfully crossed the BBB and migrated to the spinal cord lesion, where it significantly downregulated PTEN expression in neurons. As a result, axonal regeneration and neovascularization were enhanced, while microgliosis and astrogliosis were reduced. This resulted in a significant improvement in locomotor, sensory, and bladder function in rats with complete SCI.^126^

The applications of intranasal administration route are not limited to the neurological field. Betzer et al. found that EVs primarily accumulated in the lungs at 24 h post-intranasal administration, whereas EVs administered intravenously were mainly localized in the liver, lungs, and spleen,^123^ indicating that intranasal administration also holds significant potential for treating pulmonary-related diseases. However, several limitations of the intranasal administration need to be considered, including the mucociliary clearance, enzymatic degradation, and potential nasal mucosa irritation, which may affect the stability and utilization of the nucleic acid-loaded EVs. To address these challenges, the functional modification and incorporation of excipients in EVs can be explored.

## Inhalation administration

Inhalation administration is widely used for direct pulmonary drug delivery due to the lungs’ extensive vascular network, large surface area, and relatively permeable mucosal barrier. Moreover, drugs reach the pulmonary circulation bypassing first-pass metabolism by directly entering the systemic circulation through the pulmonary veins.^10^

Cheng et al. discovered that lung spheroid cell-derived exosomes (LSC-Exo) were more efficient at evading mucoadhesion compared to their liposome counterparts.^127^ Additionally, LSC-Exo maintained a higher level of exosomal mRNA and protein cargo deposition, retention, and distribution in the lung parenchyma and bronchioles.^127^ They further constructed angiotensin-converting enzyme II (ACE2)-expressed LSC-Exo, which was retained and distributed throughout the lung over time. These inhaled LSC-Exo then bound to spike (S) protein of SARS-CoV-2, leading to a significant reduction in lung inflammation and viral load, thereby effectively alleviating SARS-CoV-2 infection.^128^ Cheng et al. also developed HEK 293T cell-derived EVs loaded with IL-12 mRNA for the treatment of orthotopic lung tumors.^129^ Twenty-four hours after inhalation administration, the IL-12 mRNA-loaded EVs preferentially accumulated in the lungs, rather than in other organs such as the liver and kidneys. These EVs were subsequently internalized by tumor cells, effectively retarding tumor growth.

Through inhalation delivery by a nebulizer, the drug can be directly deposited into the respiratory tract, making this route an attractive option for pulmonary drug delivery.^130^ However, inhalation of EVs into the lungs via spontaneous breathing makes it challenging to control the dosage. Additionally, both the integrity of the EVs and the release of encapsulated drugs should be carefully considered before and after atomization to ensure their efficacy and stability.

## Conclusions and future perspectives

Nucleic acid-based therapies hold great potential for treating previously incurable diseases. However, effective and targeted delivery to specific tissues, efficient cellular internalization, and precise release at the action spatial position remain significant challenges. These difficulties arise from the large molecular size, negative charge, and susceptibility to degradation of nucleic acids. Viral vectors and chemically synthesized NPs are currently widely studied nucleic acid carriers.^8^^,^^41^ However, viral vectors face issues such as unsatisfactory targeting, cargo-loading capacity, and concerns over immunogenicity and toxicity. On the other hand, chemically synthesized NPs are limited by rapid clearance *in vivo*, low transfection efficiency, and an increased risk of immunogenicity, which hinder their clinical application. Owing to the favorable pharmacokinetics, immunological compatibility, and ability to cross physiological barriers, EVs have emerged as promising nucleic acid delivery carriers and have been tested both preclinically and clinically. For example, miR-124-loaded MSC-derived EVs have been used to ameliorate brain injury by promoting neurogenesis and have been included in clinical trials for treating ischemic stroke (NCT03384433).^131^ Moreover, a phase 1 clinical trial (NCT03608631) investigating the safety, tolerability, and preliminary efficacy of KRAS-G12D siRNA-loaded MSC-derived EVs in patients with metastatic pancreatic ductal adenocarcinoma identified its potential to remodel the tumor immune microenvironment for combination with immunotherapy.^132^

Despite these promising developments, several engineering challenges must be addressed for the successful clinical application of EVs for nucleic acid delivery, particularly when compared with synthetic nanocarriers. First, the innate tropism of EVs appears to be limited.^133^ Therefore, targeting moieties need to be incorporated on the EV surface to assist nucleic acids to reach desired tissues and cells, thus minimizing the off-target effects. In this context, possible immunogenicity and toxicity induced by surface modifications should be carefully considered. Second, the fate of EVs and cargoes within the bloodstream, their tissue distribution tropism, and subcellular fate remain to be fully investigated. These mechanistic understandings are crucial not only for optimizing nucleic acid loading and EV modification strategies but also for establishing more explicit structure-activity relationships that can be compared with those already existing for synthetic nanocarriers. Third, although EVs exhibit superior biocompatibility, their production is still restricted by low yield, batch-to-batch variability, and limited standardization of upstream cell culture and downstream isolation processes. In contrast to the well-defined compositions and scalable manufacturing of synthetic nanocarriers, EV preparations remain highly heterogeneous in terms of size, cargo, and surface protein profiles. These make it difficult to define robust quality control criteria and to fully meet regulatory expectations for reproducibility and potency. Fourth, the loading of nucleic acids into EVs remains less efficient and less controllable than those in many synthetic systems. Current endogenous and exogenous loading strategies often involve trade-offs between the loading efficiency, vesicle integrity, and scalability. Moreover, the impact of specific EV preparation methods on subsequent cargo loading (e.g., via changes in membrane rigidity, permeability, and surface composition) is not yet fully understood. Coupled with the generally lower yield and higher process complexity, these factors make EV production relatively costly compared with synthetic carriers.

Therefore, to fully unleash the potential of EVs as nucleic acid delivery vehicles, several priority tasks need to be tackled. These include developing reliable and low-immunogenicity strategies to augment EV targeting, attaining a more in-depth mechanistic comprehension of EV biodistribution, trafficking, and the fate of their cargo *in vivo*, standardizing scalable and reproducible biomanufacturing workflows to reduce heterogeneity, and enhancing loading technologies to achieve higher and more controllable cargo encapsulation. Sustained progress in these domains will establish a more robust foundation for the rational design of EV delivery systems. Owing to these advancements, EVs are well positioned to emerge as a highly competitive and potential platform for nucleic acid therapeutics, complementing and, in specific applications, outperforming current synthetic nanocarriers.

## Acknowledgments

This work was supported by the 10.13039/501100012166National Key Research and Development Program of China (2024YFA1108701 to K.Y.), 10.13039/100014718National Natural Science Foundation of China (82404564 to X.Z. and 82300948 to Q. Z.), 10.13039/501100021171GuangDong Basic and Applied Basic Research Foundation (2023A1515110428 and 2025A1515012065), Shenzhen Medical Research Fund (D2401001), 10.13039/501100017610Shenzhen Science and Technology Program (JCYJ20240813142059020). The funders did not have any role in paper design, data collection, and writing or revision of this manuscript.

## Author contributions

Q.Z. and K.Y. contributed to the conception and design of this study; X.Z. and X.L. wrote the original draft and created all the figures; Q.Z. and K.Y. revised and edited the review.

## Declaration of interests

None declared.
